# The effects of anatabine on non-invasive indicators of muscle damage: a randomized, double-blind, placebo-controlled, crossover study

**DOI:** 10.1186/1550-2783-10-33

**Published:** 2013-07-22

**Authors:** Nathaniel DM Jenkins, Terry J Housh, Glen O Johnson, Daniel A Traylor, Haley C Bergstrom, Kristen C Cochrane, Robert W Lewis,, Richard J Schmidt, Joel T Cramer

**Affiliations:** 1 Department of Nutrition and Health Sciences, University of Nebraska-Lincoln, 211 Ruth Leverton Hall, Lincoln, NE 68583-0806, USA

**Keywords:** Anatabine, Supplementation, Eccentric muscle damage, Delayed onset muscle soreness, Muscle function

## Abstract

**Background:**

Anatabine (ANA), a minor tobacco alkaloid found in the Solanaceae family of plants, may exhibit anti-inflammatory activity, which may be useful to aid in recovery from exercise-induced muscle damage. The purpose of this study, therefore, was to examine the effects of ANA supplementation on the recovery of isometric strength and selected non-invasive indicators of muscle damage.

**Methods:**

A double-blinded, placebo-controlled, crossover design was used to study eighteen men (mean ± SD age = 22.2 ± 3.1 yrs; body mass = 80.3 ± 15.7 kg) who participated in two randomly-ordered conditions separated by a washout period. The ANA condition consisted of consuming 6–12 mg anatabine per day for 10 days, while testing took place during days 7–10. The placebo (PLA) condition was identical except that the PLA supplement contained no ANA. Maximal voluntary isometric peak torque (PT) of the forearm flexors, arm circumference, hanging joint angle, and subjective pain ratings were measured before (PRE), immediately after (POST), and 24, 48, and 72 h after six sets of 10 maximal, eccentric isokinetic forearm flexion muscle actions. Resting heart rate and blood pressure were measured at PRE and 72 h in each condition.

**Results:**

For PT, hanging joint angle, arm circumference, and subjective pain ratings, there were no condition x time (p > 0.05) interactions, there were no main effects for condition (p > 0.05), but there were main effects for time (p < 0.001). There were no condition x time (p > 0.05) interactions and no main effects for condition (p > 0.05) or time (p > 0.05) for blood pressure or resting heart rate.

**Conclusions:**

ANA supplementation had no effect on the recovery of muscle strength, hanging joint angle, arm swelling, or subjective pain ratings after a bout of maximal eccentric exercise in the forearm flexors. Therefore, ANA may not be beneficial for those seeking to improve recovery from heavy eccentric exercise. Future studies should examine the effects of ANA on the pro-inflammatory cytokine responses to exercise-induced muscle damage and the chronic low-grade inflammation observed in obese and elderly individuals.

## Background

Unaccustomed eccentric exercise often results in muscle damage and delayed onset muscle soreness (DOMS). The symptoms of eccentric-induced muscle damage include loss of strength, limited range of motion, swelling, pain, and tenderness [1,2]. While the exact mechanisms responsible for these symptoms are not fully understood, the structural damage caused by eccentric exercise is characterized by disorganization of myofilaments, z-line widening and streaming [3,4], interruption of the excitation-contraction coupling process [5], and an immune response that produces an accumulation of mononuclear cells [1]. It is also possible that neural mechanisms, such as the inability to fully activate muscles, may contribute to the loss of strength following eccentric exercise [6,7]. Thus, several factors contribute to the manifestation of eccentric-induced symptoms of muscle damage and DOMS. As a result, studies have examined a variety of treatments to reduce damage or improve recovery after eccentric exercise, such as therapeutic modalities (i.e., massage, cryotherapy, and stretching), pharmacological treatments (i.e., non-steroidal anti-inflammatory drugs), and dietary supplementation.

Lund et al. [8] showed no effects of passive stretching on muscle strength or muscle pain after eccentric-induced muscle damage in the leg extensors. Tokmakidis et al. [9] demonstrated that ibuprofen (400 mg every 8 hours for 48 hrs) decreased muscle soreness at 24 h after eccentric exercise, however, there were no differences in the recovery of muscle strength or range of motion compared to placebo. In addition, Connolly et al. [10] found that tart cherry juice supplementation attenuated the losses in muscle strength and decreased muscle pain after eccentric-induced muscle damage when compared to a placebo. Consequently, treatments that may reduce inflammation can help to improve recovery or alleviate the symptoms associated with exercise-induced muscle damage.

Anatabine (ANA) is a minor alkaloid with a similar chemical structure to nicotine that is found in the tobacco plant and the Solanaceae family of plants (i.e., green tomatoes, eggplant, and peppers). Recent studies have observed anti-inflammatory effects of ANA [11,12]. For example, ANA lowered NFkB activation and limited amyloid beta production, both of which are associated with plaque deposits in the brain, in Alzheimer’s disease [11] and the over-production of brain inflammatory cytokines [12]. ANA has also been shown to prevent the production of interleukin-1 beta (IL-1β), interleukin 6 (IL-6), and tumor necrosis factor alpha (TNF-α) induced by lipopolysaccharides in human blood and in mice [12]. Theoretically, therefore, ANA may attenuate the decreases in muscle strength following eccentric-induced muscle damage by reducing inflammation and the production of pro-inflammatory cytokines, since muscle strength is commonly identified as the single best non-invasive indicator of muscle damage [2]. For instance, Beck et al. [13] demonstrated attenuated losses in muscle strength with protease supplementation following eccentric-induced muscle damage, which was explained by the potential anti-inflammatory effects of the protease supplement. Therefore, using the same experimental model as Beck et al. [13], the purpose of this study was to examine the effects of ANA supplementation on the recovery of isometric strength and selected non-invasive indicators of muscle damage. A second objective was to assess the effects of short-term ANA supplementation on heart rate and blood pressure.

We hypothesized that ANA would attenuate losses in muscular strength and improve the recovery of the hanging joint angle, relaxed arm circumference, and subjective pain ratings due to its potential anti-inflammatory properties. We also hypothesized that ANA supplementation would result in moderate decreases in blood pressure and small increases in heart rate because of its similar chemical structure to nicotine [14].

## Methods

### Participants

Twenty men (mean ± SD age = 22.4 ± 3.0 yrs; body mass = 79.4 ± 15.5 kg; height = 182.9 ± 6.5 cm) volunteered to participate in this investigation, which was approved by the university Institutional Review Board for the protection of human participants. Two men consumed less than 70% of the study product and were subsequently considered non-compliant and excluded from data analysis. Therefore, only the data from the 18 compliant men (mean ± SD age = 22.2 ± 3.1 yrs; body mass = 79.7 ± 16.1 kg; height = 182.9 ± 6.5 cm) were analyzed and reported for this study. Prior to any testing at visit 1, participants signed an informed consent form and completed a health history questionnaire. Each participant was free from current or ongoing neuromuscular diseases or musculoskeletal injuries involving the wrist, elbow, and shoulder joints. None of the participants had acute infections nor had they engaged in any upper-body resistance training during the 6 months prior to enrollment. In addition, none of the participants reported smoking, use of smokeless tobacco, or use of creatine within 9 weeks prior to enrollment. All of the participants were instructed to maintain their normal dietary habits and avoid the use of anti-inflammatory or pain medications throughout the duration of the study.

### Experimental design

**Figure 1:**
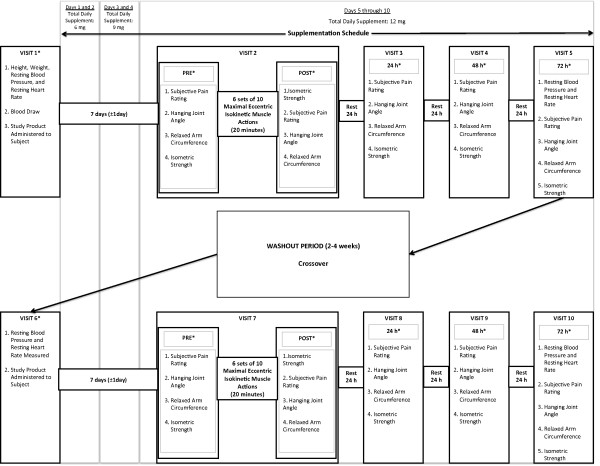
**Schematic of the testing schedule for visits 1–5 and visits 6–10.** Testing was performed before (PRE), immediately after (POST), and 24, 48, and 72 h after the eccentric exercise. *The order of tests are numbered sequentially.

This study used a randomized, double-blinded, placebo-controlled, crossover design (Figure 1). At visit 1, the participants were randomly assigned to either a supplement (anatabine, ANA) or placebo (PLA) condition based on their assigned participant number and corresponding randomization code. The participants returned to the laboratory for visit 2 seven days (± 1 day) after visit 1, and data were recorded for unilateral maximal voluntary isometric forearm flexion strength, hanging joint angle, relaxed arm circumference, and subjective pain rating. Each of these tests was performed immediately prior to (PRE), immediately following (POST), and 24, 48, and 72 h after the bout of maximal eccentric isokinetic forearm flexion exercise (Figure 1). Following a washout period of 2–4 weeks, participants returned for visit 6 to undergo either the ANA or PLA condition, whichever was not administered during visits 1–5. During the crossover (visits 6–10), the participants performed the same series of tests as visits 1–5. The dependent variables in this study were maximal voluntary isometric forearm flexion strength, hanging joint angle, relaxed arm circumference, and subjective pain rating measured during visit 2–5 and visits 7–10, while resting systolic and diastolic blood pressure and resting heart rate were only measured at baseline and 72 h (visits 1, 5, 6, and 10). Figure 1 displays the numeric order of tests performed at each visit. The independent variables in this study were condition (ANA or PLA) and time (PRE, POST, 24, 48, and 72 h), and both were within-subjects repeated measures variables.

### Supplementation

The ANA and PLA dietary supplements were administered as mint-flavored mannitol granulation lozenges. Each ANA lozenge contained 3 mg of anatabine, 834 IU vitamin A, and 66 IU vitamin D3. The PLA lozenge contained everything in the ANA lozenge except for anatabine and was identical in flavor and appearance to the ANA lozenge. The participants were given a 10 day supply of study product (ANA or PLA) at visits 1 and 6 and were instructed to self-administer the lozenges with food two or three times per day beginning after visit 1 (Figure 1).The schedule for consuming the lozenges during each 10 day period was as follows: (a) 1 lozenge at breakfast and lunch on days 1 and 2, (b) 1 lozenge at breakfast, lunch, and dinner on days 3 and 4, and (c) 2 lozenges at breakfast and 1 at lunch and dinner on days 5–10. Therefore, during the ANA condition, the participants consumed 6 mg of ANA during days 1 and 2, 9 mg during days 3 and 4, and 12 mg during days 5 through 10. The participants did not take any study product during the washout period of two to four weeks (Figure 1).

Compliance was assessed when all unused study product was returned to the laboratory at visits 5 and 10. The amount of unused product was counted and used to calculate compliance. The average compliance was (mean ± standard deviation) 95.3 ±7.7%, and compliance ranged between 74% and 104% for all 18 participants.

### Eccentric exercise protocol

During visits 2 and 7 (Figure 1), the participants completed an eccentric exercise protocol that consisted of 6 sets of 10 maximal eccentric isokinetic muscle actions of the forearm flexors at 30° s^-1^. The exercised arm (right or left) used during visit 2 was determined at visit 1 using a separate randomization, and the opposite arm was exercised at visit 7. Connolly et al. [15] reported that about of eccentric exercise in one limb does not confer a protective effect against muscle damage in the opposite limb two weeks later.

**Figure 2:**
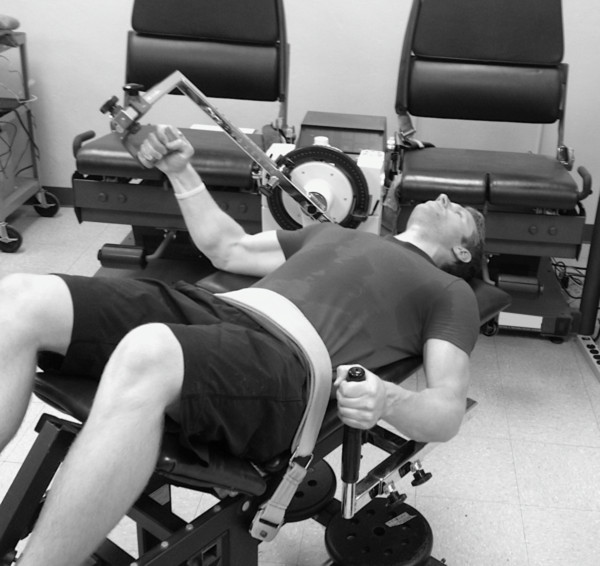
An example of participant positioning during a maximal voluntary isometric muscle action.

Participants were placed in a supine position on an upper body exercise testing bench with a strap placed around the waist to prevent excessive movement (Figure 2). The eccentric muscle actions were performed with a neutral hand position. One min of rest was given between each set. Strong verbal encouragement was provided throughout the protocol to ensure that a maximal effort was given. Following the eccentric exercise protocol, 2 min of rest was provided prior to the POST exercise assessments.

### Isometric strength

Participants were placed on an upper body exercise testing bench as previously described (Figure 2). Following a warm-up of 5 submaximal muscle actions at 50% of maximal effort, the participants performed two 6-s maximal voluntary isometric muscle actions (MVICs) of the forearm flexors separated by 2 min of rest. The MVICs were performed with a neutral hand position. Torque was recorded with a calibrated isokinetic dynamometer (Cybex 6000, CYBEX Division, LUMEX Inc., Ronkonkoma, NY). Prior to the isometric muscle actions, the limb was weighed and gravity corrected using HUMAC software (HUMAC2009, CSMi, Stoughton, MA). During the isometric muscle actions, the joint angle between the arm and forearm was set at 115° (65° from full extension), and the angle between the arm and trunk was set at 45° (45° of abduction). In order to remove any free play from the dynamometer lever arm, the investigator placed a minimal baseline pressure on the lever arm prior to the initiation of the MVICs. Careful instruction was given to each participant to ensure that they contracted as “hard and fast” as possible. The highest torque output (Nm) provided by the HUMAC software for the two MVICs was defined as the peak torque (PT) and was used for subsequent analyses.

### Hanging joint angle and relaxed arm circumference

The hanging joint angle (°) between the forearm and arm was measured using a standard goniometer (Smith and Nephew Rolyan Inc., Menomomee Falls, WI) [1,16]. For each measurement, the axis of rotation of the elbow joint was aligned with the axis of the goniometer. The proximal arm of the goniometer was aligned with the acromion process of the scapula and the distal arm was aligned with the styloid process of the ulna. Relaxed arm circumference (cm) was measured with a Gulick tape (Mabis Healthcare, Waukegan, IL) [16] at half the distance between the acromion process of the scapula and the olecranon process of the ulna. The maximum girth was determined with the arm horizontally abducted and the forearm extended. The hanging joint angle and relaxed arm circumference were always measured on the exercised arm prior to completing the MVIC, except during the POST assessments at visits 2 and 7 (Figure 1) when hanging joint angle and relaxed arm circumference were measured after the MVIC.

### Subjective pain rating

An arm pain intensity scale adapted from McHugh and Tetro [17] was used to examine the subjective pain rating in the forearm flexors of the exercised arm as described by Beck et al. [13]. The scale ranged from 0 (no pain at all) to 10 (extremely intense pain). The participants were presented the scale and asked to identify a single score for arm pain with the arm relaxed and hanging at the participant’s side. The subjective pain rating was assessed prior to MVIC, except during the POST assessments at visits 2 and 7 (Figure 1) when the subjective pain rating was assessed after the MVIC.

### Resting blood pressure and resting heart rate

The resting blood pressure and resting heart rate were measured after the participant had been sitting quietly for a period of at least 5 minutes prior to any other testing. Systolic and diastolic resting blood pressure were measured in mmHg with an aneroid sphygmomanometer(MDF Instruments, Agoura Hills, CA) and a stethoscope (Marshall Nurse Stethoscope, Riverside, IL) according to the procedures described by Housh et al. [18]. Resting heart rate was measured by palpating the radial artery at the anterior-lateral surface of the wrist in line with the base of the thumb, just medial to the styloid process of the radius. Once the pulse was located, the number of beats that occurred in 30 s was measured and multiplied by two to calculate the resting heart rate (bpm).

### Statistical analyses

Four separate two-way repeated measures analyses of variance (ANOVAs) (condition [ANA vs. PLA] x time [PRE vs. POST vs. 24 h vs. 48 h vs. 72 h]) were used to analyze PT, hanging joint angle, relaxed arm circumference, and subjective pain rating. Three separate two-way repeated measures ANOVAs (condition [ANA vs. PLA] × time [PRE vs. 72 h]) were used to analyze systolic blood pressure, diastolic blood pressure, and resting heart rate. When appropriate, follow-up analyses included one-way repeated measures ANOVAs and Bonferonni-corrected dependent samples t-tests. All statistical analyses were performed using IBM SPSS v. 21 (Chicago, IL), and a type I error rate of 5% was considered statistically significant for all comparisons.

## Results

**Figure 3:**
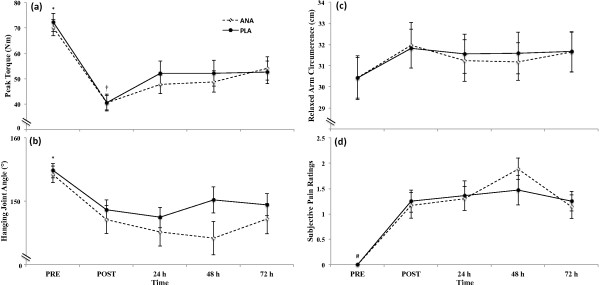
**Recovery of the non-invasive measures of muscle function.** Data presented are means ± standard error of the mean for **(a)** isometric forearm flexion strength (Nm), **(b)** hanging joint angle (°), **(c)** relaxed arm circumference (cm), and **(d)** subjective pain ratings during the supplement (dashed line, open circles; ANA) and placebo (solid line, closed circles; PLA) conditions assessed before (PRE), immediately after (POST), and 24 h, 48 h, and 72 h after a bout of maximal eccentric exercise. For the marginal means (collapsed across condition), *PRE > POST, 24 h, 48 h, and 72 h (p < 0.05); ^†^POST < PRE, 24 h, 48 h, and 72 h (p < 0.05); ^#^PRE < POST, 24 h, 48 h, and 72 h (p < 0.05).

There were no condition x time (p > 0.05) interactions, there were no main effects for condition (p > 0.05), but there were main effects for time for PT (p < 0.001), hanging arm joint angle (p < 0.001), relaxed arm circumference (p < 0.001), and subjective pain rating (p < 0.001). The marginal means for PT (collapsed across condition) decreased (p < 0.001) from PRE to POST, increased (p = 0.001) from POST to 24 h, and then plateaued (p > 0.05) from 48 h to 72 h (Figure 3a). The marginal means for hanging joint angle (collapsed across condition) decreased (p < 0.001) from PRE to POST and then did not change (p > 0.05) from POST to 72 h (Figure 3b). The marginal means for relaxed arm circumference (collapsed across condition) increased from PRE to POST (p < 0.001) and then plateaued (p > 0.05) from POST to 72 h (Figure 3c). The marginal means for subjective pain ratings (collapsed across condition) increased (p < 0.001) from PRE to POST, but did not change (p > 0.05) from POST to 72 h (Figure 3d).

**Figure 4:**
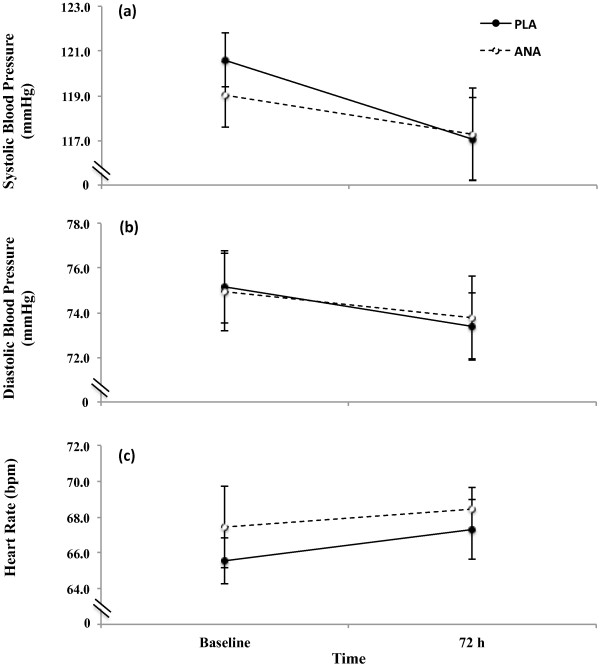
**Heart rate and blood pressure.** Data presented are means ± standard error of the mean for **(a)** systolic blood pressure (mmHg), **(b)** diastolic blood pressure (mmHg), and **(c)** heart rate (bpm) during the supplement (dashed line, open circles; ANA) and placebo (solid line, closed circles; PLA) conditions assessed at baseline (visits 1 or 6)and 72 h after the bout of maximal eccentric exercise.

There were no condition x time (p > 0.05) interactions and no main effects for condition (p > 0.05) or time (p > 0.05) for systolic blood pressure (Figure 4a), diastolic blood pressure (Figure 4b), or resting heart rate (Figure 4c).

## Discussion

The results of the present study did not support our original hypotheses that ANA would improve the recovery of PT, hanging joint angle, relaxed arm circumference, or subjective pain ratings compared to PLA in response to eccentric-induced muscle damage. The protocol used in the present study has been used to elicit muscle damage in previous studies [6,13,19,20]. For example, Beck et al. [13] demonstrated 21-43% decreases in PT of the forearm flexors, while Cockburn et al. [20] reported 15-20% decreases in leg flexion PT. The 23-44% decreases in PT observed in the present study were consistent with Beck et al. [13], but greater than Cockburn et al. [20], which may have been related to the muscle group studied. Nevertheless, Warren et al. [2] suggested that PT is the single best non-invasive indicator of muscle damage resulting from eccentric exercise, therefore, the results of the present study suggested that the magnitude of muscle damage that occurred was consistent with or greater than previous studies using the same protocol. Interestingly, these previous studies [13,20] and others [10] have also demonstrated that this muscle damage protocol has elicited decreases in PT that were sensitive to dietary supplement interventions to improve recovery. However, in the present study there were no differences between ANA and PLA conditions during the recovery of PT, hanging joint angle, relaxed arm circumference, or subjective pain rating within 72 h after eccentric exercise. Thus, our conclusion was that ANA supplementation had no effect on recovery of muscle strength, joint stiffness, arm swelling, or pain using this model of muscle damage.

Connelly et al. [10] demonstrated that 8 days of cherry juice supplementation attenuated losses in isometric forearm flexion PT following a bout of eccentric exercise when compared with a placebo. The authors [10] hypothesized that the preservation of strength was likely due to the anti-inflammatory and antioxidant properties of the cherry juice. Similarly, Beck et al. [13] showed less reductions in isometric forearm flexion PT after eccentric exercise when participants supplemented with protease enzymes compared to placebo. Beck et al. [13] hypothesized that the improved recovery may have been caused by decreases in acute inflammation as a result of improved return of interstitial fluid to the bloodstream and decreased production of prostaglandins with protease supplementation. In contrast, Rawson et al. [21] failed to show an effect of creatine supplementation on the recovery of isometric forearm flexion strength following eccentric exercise. The authors [21] hypothesized that the mechanical loads placed on the muscle were too great for creatine to display any membrane-stabilizing effects. Likewise, the results of the current study indicated that there were no differences between the ANA and PLA conditions for the decreases in and recovery of PT following the eccentric exercise protocol. It is possible, however, that ANA may reduce inflammation under other physiological conditions. For example, obesity and aging are associated with increased baseline systemic inflammation that is driven by greater secretion of pro-inflammatory cytokines compared to young, healthy individuals [22-24]. Future studies should examine the effects of ANA on the inflammation associated with obesity and aging.

Studies on the effects of ANA in animal models [11,12] have demonstrated that ANA exerts anti-inflammatory effects via inhibition of Signal Transducer and Activator of Transcription 3 (STAT3) and NFkB phosphorylation. It has also been shown [12] that ANA may reduce pro-inflammatory cytokine (i.e. TNF-α, IL-6, and IL-1β) production. Despite evidence that ANA has anti-inflammatory effects [12], as well as evidence that dietary supplementation may improve the recovery of strength after eccentric-induced muscle damage [10,13], ANA supplementation had no discernable effect on PT or the other measures of muscle function following eccentric-induced muscle damage. It is possible that the pathways by which ANA may elicit anti-inflammatory effects may not influence the recovery of muscle function following eccentric-induced muscle damage. Future studies should investigate the effects of ANA on pro-inflammatory cytokine responses after eccentric exercise.

Eccentric-induced muscle damage may cause muscle shortening without neural activation as a result of calcium leakage from the sarcoplasmic reticulum [1]. It has also been suggested [25] that the movement of cells and fluid from the circulation into the interstitial spaces surrounding muscle fibers results in inflammation and edema after eccentric exercise. Therefore, previous studies have utilized the hanging joint angle [10,13] and relaxed arm circumference [1,13,16], respectively, as markers of eccentric-induced muscle damage. However, when these indices have been used to assess the ability of dietary supplements to enhance recovery after heavy eccentric exercise, they have been unable to detect a treatment effect [10,13]. Muscle soreness, assessed by the subjective pain rating in the present study, is one of the most commonly used measures of exercise-induced muscle injury [2]. However, Warren et al. [2] suggested that soreness correlates poorly with muscle function. In the current study, the patterns of recovery for hanging joint angle, relaxed arm circumference, and subjective pain ratings were similar in the ANA and PLA conditions (Figure 3b). Therefore, the lack of effect of supplementation on the hanging joint angle and relaxed arm circumference in these studies [10,13], the poor correlation between muscle function and soreness [2], and the results of the present study have collectively suggested that these indicators of muscle damage may not be sensitive to dietary supplement interventions to improve recovery from eccentric-induced muscle damage. Future studies may wish to consider these findings when selecting outcome variables for assessing the efficacy of dietary supplementation for reducing muscle damage.

A secondary objective of this study was to examine the effects of 10 days of ANA dietary supplementation on resting heart rate and blood pressure. As a minor alkaloid with a similar chemical structure to nicotine, we hypothesized that ANA would cause moderate decreases in systolic and diastolic blood pressure and small increases in resting heart rate. Previous studies [14,26,27] have shown that acute nicotine exposure causes an increase heart rate and blood pressure through stimulation of the sympathetic nervous system. Minami et al. [28] showed that smoking cessation caused a reduction in heart rate, which implied that chronic nicotine use may elevate heart rate. However, cross sectional studies [26,29] have shown that systolic and diastolic blood pressures are lower in smokers than in non-smokers. The results of the present study indicated that, over a period of 10 days, ANA had no effect on heart rate or blood pressure compared to placebo. Thus, ANA supplementation may be safe regarding short-term use (10 days) on resting heart rate and blood pressure. However, future studies may wish to examine the acute and chronic effects of ANA consumption on blood pressure and heart rate to further discern its safety.

## Conclusions

In conclusion, ANA supplementation had no effect on the recovery of muscle strength, hanging joint angle, arm swelling, or subjective pain ratings after a bout of maximal eccentric exercise in the forearm flexors. Therefore, ANA may not be beneficial for those seeking to improve recovery from heavy exercise training or competition. Future studies, however, should examine the effects of ANA on the pro-inflammatory cytokine responses to eccentric-induced muscle damage. It is also possible that, due to the pathways by which ANA exerts its anti-inflammatory properties, ANA supplementation may have an effect on chronic, low-grade inflammation such as the inflammation observed in obese and elderly individuals.

## Abbreviations

ANA: Anatabine; ANOVA: Analysis of variance; DOMS: Delayed onset muscle soreness; IL-1β: Interleukin-1 beta; IL-6: Interleukin 6; MVICs: Maximal voluntary isometric muscle actions; PLA: Placebo; PT: Peak torque; STAT3: Signal Transducer and Activator of Transcription 3; TNF-α: Tumor necrosis factor alpha.

## Competing interests

TJH and JTC are the principle or co-investigators of currently-funded research or service contracts at the University of Nebraska-Lincoln with Rock Creek Pharmaceuticals, Abbott Nutrition, General Nutrition Center, and Stepan Lipid Nutrition. NDMJ, DAT, KCC, HCB, and RWL Jr. declare that they have no competing interests.

## Authors’ contributions

NDMJ was the primary manuscript writer, and carried out data acquisition, data analysis and data interpretation. DAT, KCC, HCB, and RWL Jr. were significant contributors to data acquisition and were important manuscript reviewers/revisers. GOJ, RJS, and TJH were significant manuscript reviewers/revisers and were substantial contributors to conception and design of this study. JTC was the primary manuscript reviewer/reviser, a substantial contributor to concept and design, and contributed to data analysis and interpretation. All authors read and approved the final manuscript.

## Authors’ information

NDMJ, KCC, and HCB are currently Ph.D. students and research assistants in the Human Performance Laboratory in the Department of Nutrition and Health Sciences at the University of Nebraska-Lincoln. DAT and RWL Jr. were research assistants in the Human Performance Laboratory at the time of data acquisition and manuscript preparation. GOJ is a professor-emeritus in the Department of Nutrition and Health Sciences at UNL. JTC, TJH, and RJS are faculty in the Department of Nutrition and Health Sciences at UNL and mentor graduate students in the Human Performance Laboratory.
